# In-Depth Molecular Profiling Specifies Human Retinal Microglia Identity

**DOI:** 10.3389/fimmu.2022.863158

**Published:** 2022-03-18

**Authors:** Julian Wolf, Stefaniya Boneva, Dennis-Dominik Rosmus, Hansjürgen Agostini, Günther Schlunck, Peter Wieghofer, Anja Schlecht, Clemens Lange

**Affiliations:** ^1^ Eye Center, Medical Center, Faculty of Medicine, University of Freiburg, Freiburg, Germany; ^2^ Institute of Anatomy, Leipzig University, Leipzig, Germany; ^3^ Cellular Neuroanatomy, Institute of Theoretical Medicine, Medical Faculty, University of Augsburg, Augsburg, Germany; ^4^ Institute of Anatomy and Cell Biology, Julius-Maximilians-University Wuerzburg, Wuerzburg, Germany; ^5^ Ophtha-Lab, Department of Ophthalmology at St. Franziskus Hospital, Muenster, Germany

**Keywords:** retinal microglia, brain microglia, human, mouse, monocytes, RNA sequencing, age-related macular degeneration (AMD), diabetic retinopathy (DR)

## Abstract

Microglia are the tissue-resident macrophages of the retina and brain, being critically involved in organ development, tissue homeostasis, and response to cellular damage. Until now, little is known about the molecular signature of human retinal microglia and how it differs from the one of brain microglia and peripheral monocytes. In addition, it is not yet clear to what extent murine retinal microglia resemble those of humans, which represents an important prerequisite for translational research. The present study applies fluorescence-activated cell sorting to isolate human retinal microglia from enucleated eyes and compares their transcriptional profile with the one of whole retinal tissue, human brain microglia as well as classical, intermediate and non-classical monocytes. Finally, human retinal microglia are compared to murine retinal microglia, isolated from *Cx3cr1* ^GFP/+^ mice. Whereas human retinal microglia exhibited a high grade of similarity in comparison to their counterparts in the brain, several enriched genes were identified in retinal microglia when compared to whole retinal tissue, as well as classical, intermediate, and non-classical monocytes. In relation to whole retina sequencing, several risk genes associated with age-related macular degeneration (AMD) and diabetic retinopathy (DR) were preferentially expressed in retinal microglia, indicating their potential pathophysiological involvement. Although a high degree of similarity was observed between human and murine retinal microglia, several species-specific genes were identified, which should be kept in mind when employing mouse models to investigate retinal microglia biology. In summary, this study provides detailed insights into the molecular profile of human retinal microglia, identifies a plethora of tissue-specific and species-specific genes in comparison to human brain microglia and murine retinal microglia, and thus highlights the significance of retinal microglia in human retinal diseases and for translational research approaches.

## Introduction

Microglia are the tissue-resident macrophages of the retina and brain that originate from the extra-embryonic yolk sac, develop in a PU.1- and IRF8-dependent manner (1, 2) and have essential functions in organ development, tissue homeostasis, and response to cellular damage (1, 3–8). In recent years, major advances in sequencing technology have demonstrated that microglia are critically involved in the pathogenesis of several brain and retinal diseases, such as Alzheimer’s disease, Parkinson’s disease and multiple sclerosis (9–11) as well as diabetic retinopathy (DR) (12) and age-related macular degeneration (AMD) (7, 8, 13). Consequently, microglia arouse growing interest as a new and promising therapeutic target in neovascular, inflammatory and neurodegenerative diseases (6–8, 14–16). The transcriptional profile of human and murine brain microglia has been deciphered in great detail, substantially improving our understanding of the role of microglia in human brain disease (9). However, the transcriptional profile of human retinal microglia, its comparability with murine microglia, and their role in retinal disease are still largely unknown.

The present study applies fluorescence-activated cell sorting (FACS) to isolate human retinal microglia from enucleated eyes and compares their transcriptional profile with that of whole retinal tissue, as well as classical, intermediate and non-classical monocytes. Since most of our knowledge on retinal microglia is currently based on animal models, this study also compared human to murine retinal microglia providing a resource for future research. The results offer detailed insights into the transcriptional profile of human retinal microglia, suggest their involvement in human retinal diseases such as AMD or DR, and identify several conserved markers but also species-specific transcripts between human and mice, paving the way for potential translational and immunomodulatory therapeutic approaches.

## Material and Methods

### Patients and Tissue Preparation

Six patients with melanoma of the iris, ciliary body or choroid undergoing enucleation at the University Eye Hospital Freiburg between 2018 and 2020 were included in this study. None of the six patients suffered from AMD or DR. Immediately after enucleation, the eye ball was divided into two halves, one containing the tumor and a tumor-free one. The tumor-comprising part was processed for routine ophthalmopathologic examination, whereas the healthy part was used to collect retinal tissue from the macular region and periphery. Immediately after enucleation, retinal tissue was separated from RPE/choroidal tissue in the operating theatre, and then instantly placed on dry ice. Unfixed retinal tissue (on ice) was then used for isolation of retinal microglia within four hours after enucleation. RNA was subsequently extracted from isolated cells and stabilized in RNA protection buffer. In addition, whole blood was obtained from three of these patients (patients #1, 2 and 5) as well as from three healthy age- and sex-matched individuals (patients #7, 8 and 9) and processed within two hours for isolation of classical, intermediate and non-classical monocytes. Additionally, transcriptional profiles of retinal pigment epithelium (RPE) and choroid tissue, collected from enucleated eyes and previously published by our group (17) were used to analyze the expression of AMD and DR risk genes. The study protocol was reviewed and approved by the local ethics committee (University Freiburg, Germany, approval number 20-1165). Written informed consent was obtained from each patient.

### Mice

The transcriptional profile of murine retinal microglia previously published by our group (18) was re-analyzed and compared to data from human retinal microglia acquired in this study. In brief, immediately after enucleation, eyes were dissected in ice-cold PBS to isolate the retinae. Unfixed retinal tissue (on ice) was then used for isolation of retinal microglia as CD45^low^ CD11b^+^ CX_3_CR1^+^ Ly6C^-^ Ly6G^-^ cells from at least two-year-old *Cx3cr1* ^GFP/+^ mice, as described before (18).

### Fluorescence-Activated Cell Sorting

Human retinal microglia were isolated from retinal tissue of the macular region and classical, intermediate and non-classical monocytes from whole blood using fluorescence-activated cell sorting (FACS), as previously described (19). To generate a single cell suspension, human retinal tissue was dissociated by resuspension using pipettes. After filtering cell solutions through a 70 μm cell strainer, 0.5 μL of Fixable Viability Dye (eFluor™ 780, eBioscience™) per 1 mL of cell solution was added. In what follows, anti-CD16/CD32 Fc block (BD Pharmingen) was performed at 4°C for 20 min at a concentration of 1:200 to avoid unspecific binding. Cells were stained for CD45 (BV421, anti-human, 1:100, BioLegend), CD11b (FITC, anti-human, 1:100, BioLegend), CX_3_CR1 (PE-Cy7, anti-human, 1:200, BioLegend) and CCR2 (PerCP/Cy5.5, anti-human, 1:200, BioLegend). To exclude any potential contamination with blood-derived monocytes due to possible surgically induced micro bleedings, we further used the Anti-Human Mature Macrophages (MatMac) antibody, an ED2-like (ectodermal dysplasia 2) marker for resident macrophages, which is absent in monocytes (20–23) (eFluor660, anti-human, 1:100, and eBioscience). For isolation of monocytes, Peripheral Blood Mononuclear Cells (PBMC) were separated from the buffy coat using density gradient centrifugation (Ficoll-Hypaque, Anprotec) according to manufacturer’s instructions. PBMCs were stained for CD45 (BV421, anti-human, 1:100, BioLegend), CD11b (FITC, anti-human, 1:100, BioLegend), CX_3_CR1 (PE-Cy7, anti-human, 1:200, BioLegend), CCR2 (PerCP/Cy5.5, anti-human, 1:200, Biolegend), CD14 (PE, anti-human, 1:200, BioLegend) and CD16 (BV711, anti-human, 1:200, BioLegend). After an incubation step of 20 min at 4°C, cells were resuspended in FACS buffer and processed for sorting on the FACS Fusion (BD Pharmingen). Viable single cells (doublet exclusion) were sorted into RNA Protect Cell Reagent (QIAGEN) at 2–8°C until sequencing was performed, as previously described (19). Retinal microglia were isolated as CD45^+^ CD11b^+^ CX_3_CR1^+^ MatMac^+^ CCR2^-^ cells. Monocytes were isolated as CD45^+^ CD11b^+^ CX_3_CR1^+^ CD14^++^ CD16^-^ (classical monocytes), CD45^+^ CD11b^+^ CX_3_CR1^+^ CD14^++^ CD16^+^ (intermediate monocytes) and CD45^+^ CD11b^+^ CX_3_CR1^+^ CD14^-^ CD16^++^ cells (non-classical monocytes).

### Preparation of Whole Retinal Tissue for Bulk RNA Sequencing

In addition, whole retinal tissue from the macular region as well as from the periphery was prepared for bulk RNA sequencing. In brief, immediately after enucleation, retinal tissue was separated from RPE/choroidal tissue in the operating theatre, and then instantly placed on dry ice. To prepare the tissue for whole retina RNA sequencing, it was slowly thawed on ice and then fixed in formalin and embedded in paraffin (FFPE) according to routine protocols, as previously described (17, 24). Ten to fifteen serial FFPE sections of 4 µm thickness from each sample were cut and then stored in tubes prior to RNA extraction, as previously reported (17).

### Total RNA Extraction

Total RNA was extracted from FACS-sorted cells stabilized in RNAprotect buffer according to the “Purification of total RNA from animal and human cells” protocol of the RNeasy Plus Micro Kit (QIAGEN, Hilden, Germany). In brief, cells were stored and shipped in RNAprotect buffer at 2-8°C. After pelleting by centrifugation for 5 minutes at 5,000 x g, RNAprotect was replaced by 350 µl RLT Plus buffer and the samples were homogenized by vortexing for 30 seconds. Genomic DNA contamination was removed by using gDNA Eliminator spin columns. Next, one volume of 70% ethanol was added and the samples were applied to RNeasy MinElute spin columns followed by several wash steps. Finally, the total RNA was eluted in 12 μl of nuclease-free water. Purity and integrity of RNA were assessed on the Agilent 2100 Bioanalyzer with the RNA 6000 Pico LabChip reagent set (Agilent, Palo Alto, CA, USA). RNA isolation from FFPE retinal specimens was carried out as previously described (17, 24). Briefly, total RNA was extracted from FFPE samples using the Quick-RNA FFPE Kit (Zymo Research, Irvine, California). Following DNAse I digestion using the Baseline-ZERO Kit (Epicentre, Madison, Wisconsin), the RNA concentration was quantified using the Qubit RNA HS Assay Kit on a Qubit Fluorometer (Life Technologies, Carlsbad, California). RNA quality was determined *via* the RNA Pico Sensitivity Assay on a LabChip GXII Touch (PerkinElmer, Waltham, Massachusetts).

### RNA Sequencing

The SMARTer Ultra Low Input RNA Kit for Sequencing v4 (Clontech Laboratories, Inc., Mountain View, CA, USA) was used to generate first strand cDNA from approximately 1 ng total-RNA. Double-stranded cDNA was amplified by LD PCR (12 cycles) and purified *via* magnetic bead clean-up. Library preparation was carried out as described in the Illumina Nextera XT Sample Preparation Guide (Illumina, Inc., San Diego, CA, USA). 150 pg of input cDNA were tagmented (tagged and fragmented) by the Nextera XT transposome. The products were purified and amplified *via* a limited-cycle PCR program to generate multiplexed sequencing libraries. For the PCR step, 1:5 dilutions of index 1 (i7) and index 2 (i5) primers were used. The libraries were quantified using the KAPA Library Quantification Kit - Illumina/ABI Prism User Guide (Roche Sequencing Solutions, Inc., Pleasanton, CA, USA). Equimolar amounts of each library were sequenced on a NextSeq 500 instrument controlled by the NextSeq Control Software (NCS) v2.2.0, using two 75 Cycles High Output Kits with the dual index, single-read (SR) run parameters. Image analysis and base calling were done by the Real Time Analysis Software (RTA) v2.4.11. The resulting.bcl files were converted into.fastq files with the bcl2fastq v2.18 software. RNA extraction, library preparation and RNA sequencing were performed at the Genomics Core Facility “KFB - Center of Excellence for Fluorescent Bioanalytics” (University of Regensburg, Regensburg, Germany; www.kfb-regensburg.de). Retinal FFPE samples were sequenced using the 3’ RNA sequencing method Massive Analysis of cDNA Ends (MACE) (17, 25), which allows sequencing of FFPE samples with high accuracy and without a significant impact of FFPE processing (26). Briefly, barcoded libraries comprising unique molecule identifiers were sequenced on the NextSeq 500 (Illumina) with 1 × 75 bp. PCR bias was removed using unique molecular identifiers.

### Bioinformatics

Sequencing data (fastq-files) were uploaded to the Galaxy web platform (usegalaxy.eu) (27), as previously described (25). Quality control was performed with FastQC (Galaxy Version 0.72, http://www.bioinformatics.babraham.ac.uk/projects/fastqc/last access on 03/01/2021). Reads were mapped to the human or mouse reference genome (Gencode, human: GRCh38.p13, all regions, release 37; mouse: GRCm38, all regions, release 25) with RNA STAR (Galaxy Version 2.7.7a) (28) using the corresponding Gencode main annotation file. Two BAM files for each sample (one for each lane) were combined in one BAM file per sample using Merge BAM Files (Galaxy Version 1.2.0). Reads mapped to the reference genome were counted with featureCounts (Galaxy Version 2.0.1) (29) using the aforementioned annotation files. The outputs of featureCounts were imported to RStudio (Version 1.4.1103, R Version 4.0.3). Gene symbols and orthologous genes (“one2one”) were determined based on the ENSEMBL database (human and mouse: release 103, download on 03/23/2021) (30). Genes with 0 reads in all samples were removed from the analysis. After principle component analysis (31), normalized reads and differential gene expression were calculated using the R package DESeq2 (version 1.30.1) with default parameters (Benjamini-Hochberg adjusted p-values) (31). Transcripts with log2 fold change (log2FC) > 2 or < -2 and adjusted p-value < 0.05 were considered as differentially expressed genes (DEG). Genes which were significantly upregulated in retinal microglia in comparison to all other groups (log2FC > 2 and adjusted p-value < 0.05 for each comparison) were defined as predominantly or preferentially expressed in retinal microglia. Heatmaps were created with the R package ComplexHeatmap (version 2.6.2) (32). Gene ontology analysis and its visualization with dotplots and cnetplots was performed using the R package clusterProfiler (version 3.18.1) (33). Other data visualization was done using the ggplot2 package (version 3.3.3) (34). To compare the transcriptional profiles of retinal microglia with the one of brain microglia, published RNA sequencing raw data (35) were reanalyzed (GEO accession: GSE99074, n=7 samples with origin Netherlands) using the methods described above. These 7 samples were from patients similar in age to the patients in our study (brain microglia: mean age: 76.0, min: 56.0, max: 102.0, sd: 16.8; retinal microglia: 69.2, min: 53.4, max: 93.8, sd: 17.7). In order to improve comparability of expression between both studies, the percentile of mean of normalized reads was calculated for each gene. To explore the specificity of expression profiles, known cell type-specific marker genes for different immune cell populations were examined (9, 36). In addition, expression levels of the most specific marker genes, known from single-cell RNA sequencing (scRNA-Seq) of human retinal tissue (36), of potentially contaminating cells, such as photoreceptors, retinal ganglion cells, bipolar cells, and endothelial cells, were studied. In addition, published scRNA-Seq data of human retinal (37) (data from Supplementary Table 3) as well as RPE/choroidal tissue (38) (data from SI Data 1) were reanalyzed to determine retinal microglia specificity in comparison to all other retinal or RPE/choroidal cells at single cell resolution. Genes were considered retinal microglia-specific when expression in retinal microglia was significantly higher than in all other retinal cell types (fold change > 0, adjusted p < 0.05 and mean expression in retinal microglia > mean expression in each retinal cell type). Genes associated with risk variants of age-related macular degeneration and diabetic retinopathy were determined based on the Genome Wide Association Study Catalog (39) (only genes with EntrezID, download on 04/18/2021).

## Results

### Patient Characteristics

Nine patients with a mean age of 68.9 ± 15.5 years including three females and six males were enrolled in this study. In six patients, enucleation was performed due to melanoma of the iris (n = 1), ciliary body (n = 2), or choroid (n = 3). Retinal microglia as well as classical, intermediate and non-classical monocytes were isolated from retinal tissue or whole blood using fluorescence-activated cell sorting (FACS) and processed for RNA sequencing. In addition, sequencing was performed on whole retinal tissue from the same six eyes. One microglia sample (patient #2) was excluded due to a significant monocyte contamination, as evident by high expression levels of monocyte and low expression levels of microglia marker genes in the primary data assessment (data not shown). Demographic data of all patients are summarized in **Table 1**.

**Table 1:** Demographics.

Patient-ID	Age	Sex	Diagnosis	Tissue
1	53.4	female	iris melanoma	retina retinal microglia classical monocytes intermediate monocytes non-classical monocytes
2	51.9	male	choroidal melanoma	retina classical monocytes intermediate monocytes non-classical monocytes
3	81.8	male	choroidal melanoma	retinal microglia
4	93.8	female	ciliary body melanoma	retina retinal microglia
5	55.8	male	choroidal melanoma	retinal microglia classical monocytes
6	61.4	male	ciliary body melanoma	retina retinal microglia
7	60.2	male	naevus of eyelid	classical monocytes intermediate monocytes non-classical monocytes
8	84.2	male	chronic blepharitis	classical monocytes intermediate monocytes non-classical monocytes
9	77.3	female	nasolacrimal duct obstruction	classical monocytes intermediate monocytes non-classical monocytes

### Quality of Expression Profiles

The expression profiles of any two patients resulted in mean Pearson correlation coefficients of 0.91 (min: 0.83, max: 0.96) for retinal microglia, 0.97 (min: 0.91, max: 0.99) for classical monocytes, 0.99 (min: 0.98, max: 0.99) for intermediate monocytes, 0.88 (min: 0.68, max: 0.98) for non-classical monocytes and 0.92 (min: 0.83, max: 0.99) for retinal tissue, indicating high similarities of expression profiles between different patients within all 5 groups (**Supplementary Figure 1A**). In addition, high specificity of expression profiles was confirmed analyzing known cell type-specific marker genes (9, 36, 40, 41) (**Supplementary Figure 1B**). Low to absent expression of photoreceptor-, retinal ganglion cell-, bipolar cell-, and endothelial cell-specific genes was detected in all four immune cell populations, indicating a low proportion of contaminating cells (**Supplementary Figure 1B**).

### Transcriptional Characterization of Human Retinal Microglia

**Figure 1:**
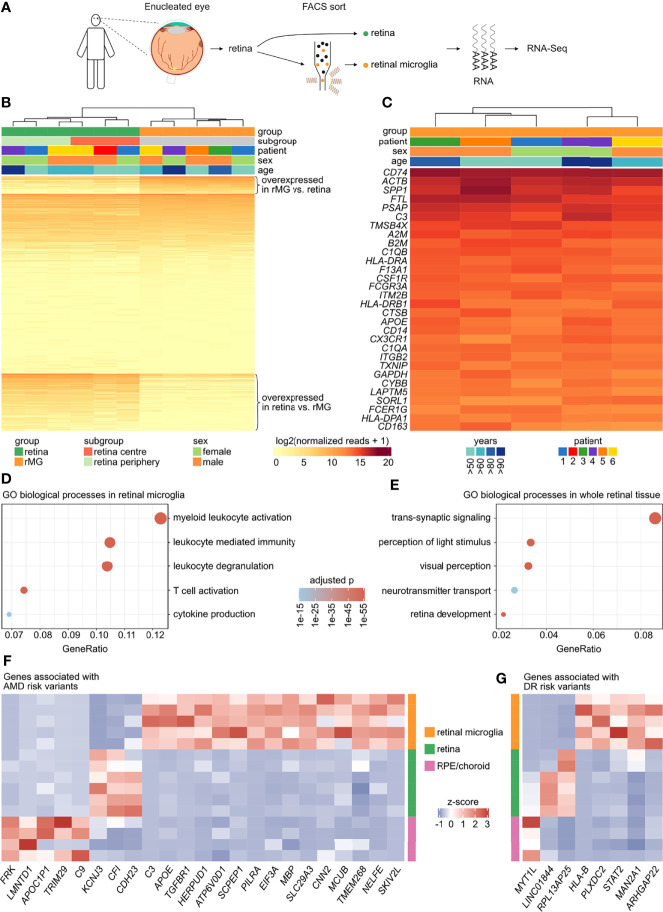
Transcriptional characterization of human retinal microglia. **(A)** Retinal tissue as well as FACS-isolated retinal microglia (CD45^+^CD11b^+^CX_3_CR1^+^MatMac^+^CCR2^-^) were analyzed by RNA sequencing. **(B)** Heatmap comparing the expression profile of 34,605 genes expressed in whole retinal tissue in comparison to sorted retinal microglia. Enriched transcripts (logFC > 2 and adjusted p < 0.05) in retinal microglia (n = 2,433) as well as in retina (n = 7,798) are shown at the top or the bottom of the heatmap, respectively. The genes with similar expression in both groups are shown in the center (n = 24,374). Genes are ordered according to mean expression in the respective group. rMG = retinal microglia. **(C)** Heatmap visualizing the top 30 genes of the retinal microglia gene signature (from B). **(D, E)** Dotplots visualizing the top five Gene ontology (GO) biological processes in retinal microglia **(D)** and in whole retinal tissue **(E)**. The GeneRatio represents the proportion of associated genes to the total number of overexpressed genes. The adjusted p-value of each GO term is shown by color. **(F, G)** Heatmaps visualizing the expression of risk genes associated with age-related macular degeneration (AMD) **(F)** or diabetic retinopathy (DR) **(G)**, which were enriched in retinal microglia, retina or retinal pigment epithelium (RPE)/choroid tissue (logFC > 2 and adjusted p < 0.05 for both comparisons each). The z-score represents a gene’s expression in relation to its mean expression by standard deviation units (red: upregulation, blue: downregulation).

Immediately after enucleation, extracted retinal tissue was divided into two parts. One part was used for whole retinal tissue sequencing and the other one for FACS-isolation of retinal microglia (CD45^+^CD11b^+^CX_3_CR1^+^MatMac^+^CCR2^-^) (**Figure 1A**). Transcriptional profiling revealed that retinal microglia expressed several brain microglia markers such as *TREM2* (triggering receptor expressed on myeloid cells 2), *P2RY12* (purinergic receptor P2Y12), and *TMEM119* (transmembrane protein 119) confirming their myeloid cell origin (**Supplementary Figure 1B**). Comparing the transcriptional profiles of retinal microglia and whole retinal tissue resulted in a total of 34,605 expressed genes, 2,433 of which were enriched in retinal microglia, whereas 7,798 were higher expressed in retinal tissue (**Figure 1B**). The expression profile of the top 30 overexpressed genes in the retinal microglia is visualized in **Figure 1C**, indicating no significant age- or sex-dependent variations. The top five of these genes were *CD74* (CD74 molecule), *ACTB* (Actin Beta), *SPP1* (Secreted Phosphoprotein 1), *FTL* (Ferritin Light Chain) and *PSAP* (Prosaposin) (**Figure 1C**). Comparing top expressed genes between retinal and brain microglia (35) revealed a high degree of similarity. The top 30 genes in retinal microglia were expressed at 99.0 percentile in brain microglia (minimum: 89.9, maximum: 100.0), while the top 30 genes in brain microglia were expressed at 97.5 percentile in retinal microglia (minimum: 80.2, maximum: 100.0). Genes with the highest similarity between retinal and brain microglia were *CD74* (retina: 100.0 percentile, brain: 99.97 percentile), *SPP1* (retina: 99.99, brain: 99.99), *ACTB* (retina: 100.0, brain: 99.97), *FTL* (retina: 99.99, brain: 99.88) and *C3* (retina: 99.98, brain: 100.0). However, some notable differences between the expression profiles of retinal and brain microglia were also observed, including *RHO* (rhodopsin, retina: 96.70 percentile, brain: 7.67 percentile, adjusted p < 2.6e-28), *CD38* (CD38 molecule, retina: 81.36, brain: 14.45, adjusted p < 1.9e-12), and *CD52* (CD52 molecule, retina: 74.16, brain: 19.31, adjusted p < 3.0e-8) being enriched in retinal microglia as well as *NEXMIF* (neurite extension and migration factor, retina: 15.45, brain: 65.88, adjusted p < 3.1e-23), *DOK6* (docking protein 6, retina: 0.0, brain: 55.17, adjusted p < 4.5e-18), and *DOCK3* (dedicator of cytokinesis 3, retina: 20.93, brain: 73.51, adjusted p < 9.9e-34) being upregulated in brain microglia.

Gene Ontology (GO) analysis revealed that the enriched genes in human retinal microglia contributed most significantly to biological processes such as myeloid leukocyte activation (GO:0002274), leukocyte degranulation (GO:0043299), leukocyte mediated immunity (GO:0002444), T cell activation (GO:0042110) and cytokine production (GO:0001819) (**Figure 1D**). The enriched genes in retinal tissue were most significantly enriched in processes like trans-synaptic signaling (GO:0099537), perception of light stimulus (GO:0050953), visual perception (GO:0007601), retina development (GO:0060041) and neurotransmitter transport (GO:0006836) (**Figure 1E**).

Having characterized the transcriptional profile of human retinal microglia in combination with parallel whole retina sequencing offered the opportunity to analyze relative expression levels of risk genes associated with age-related macular degeneration (AMD) and diabetic retinopathy (DR) (9, 39). In addition, the expression profiles of retinal pigment epithelium (RPE) and choroid tissue, previously published by our group (17), were also employed for this purpose. Of a total of 158 AMD risk genes, 139 (88.0%) were expressed in at least one of the analyzed tissues. Interestingly, 15 AMD-associated risk genes such as *C3* (Complement C3), *APOE* (Apolipoprotein E) and *TGFBR1* (Transforming Growth Factor Beta Receptor 1), were predominantly expressed in retinal microglia, whereas only three and five risk genes were preferentially expressed in retinal (e.g. *CFI* (Complement Factor I)) or RPE/choroid tissue (e.g. *C9* (Complement C9)), respectively (**Figure 1F**). Reanalysis of published scRNA-Seq data of human retinal (37) and RPE/choroid tissue (38) revealed that *C3*, *APOE*, *TGFBR1*, *PILRA* (Paired Immunoglobin Like Type 2 Receptor Alpha) and *SLC29A3* (Solute Carrier Family 29 Member 3) were mainly expressed in retinal microglia compared to 28 other retinal and RPE/choroid cell types, with however *C3* being also expressed in choroidal fibroblasts, *APOE* in retinal glia cells, and *PILRA* in choroidal macrophages (**Supplementary Figure 2B**). Of note, *C3*, *APOE* and *TGFBR1* were also strongly expressed in retinal microglia when compared to all three subtypes of monocytes (**Supplementary Figure 2A**), suggesting that these genes are predominantly expressed in resident microglia compared to potential infiltrating monocytes in AMD. Regarding DR-associated risk genes, 89.8% (n = 97) were expressed in at least one of the analyzed tissues. There were 5 DR-associated risk genes such as *HLA-B* (Major Histocompatibility Complex, Class I, B), *PLXDC2* (Plexin Domain Containing 2) and *ARHGAP22* (Rho GTPase Activating Protein 22), which were enriched in retinal microglia, whereas only two risk genes were preferentially expressed in retinal and one risk gene in RPE/choroid tissue (**Figure 1G**). Reanalysis of the above mentioned scRNA-Seq data demonstrated that *PLXDC2* and *ARHGAP22* were mainly expressed in retinal microglia compared to 28 other retinal and RPE/choroid cell types, with however *PLXDC2* being also expressed in choroidal schwann cells, and *ARHGAP22* in choroidal macrophages and cones (**Supplementary Figure 2D**). Interestingly, *PLXDC2* and *ARHGAP22* were as well strongly expressed in retinal microglia in comparison to all three subtypes of monocytes (**Supplementary Figure 2C**). In conclusion, these results indicate that several risk genes for AMD and DR are preferentially expressed in retinal microglia than in other analyzed ocular tissues, which may indicate their role in disease occurrence and progression.

### Identification of Human Retinal Microglia-Enriched Genes

**Figure 2:**
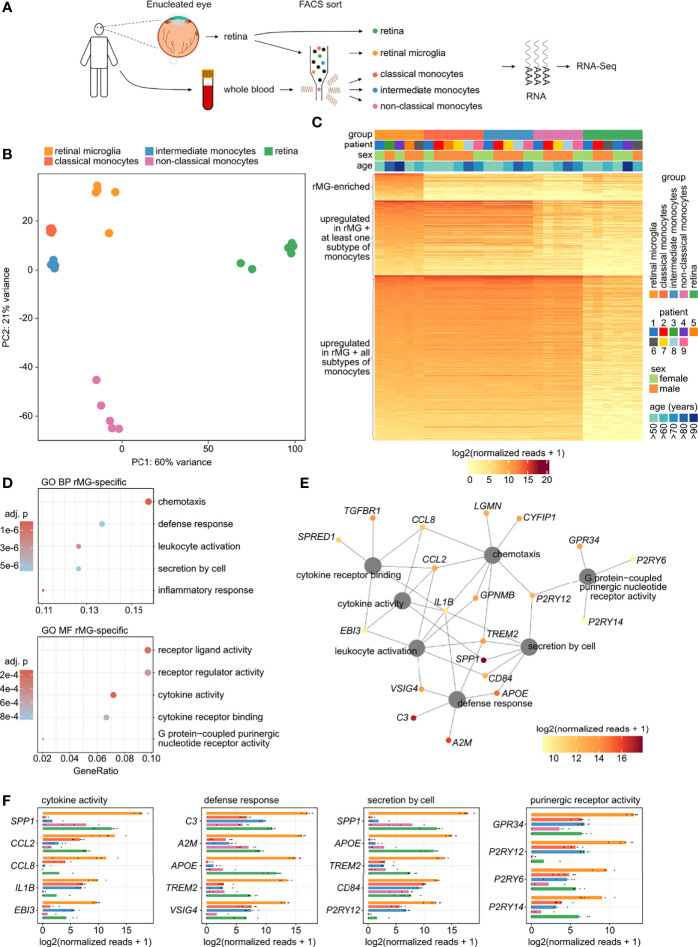
Identification of human retinal microglia-enriched genes. **(A)** Experimental setup: retinal tissue was collected from six enucleated eyes and whole blood was obtained from six patients. Retinal tissue, retinal microglia (CD45^+^CD11b^+^CX_3_CR1^+^MatMac^+^CCR2^-^), as well as classical (CD45^+^CD11b^+^CX_3_CR1^+^CD14^++^CD16^-^), intermediate (CD45^+^CD11b^+^CX_3_CR1^+^CD14^++^CD16^+^), and non-classical monocytes (CD45^+^CD11b^+^CX_3_CR1^+^CD14^-^CD16^++^) were isolated by fluorescence-activated cell sorting (FACS) followed by RNA sequencing. **(B)** Principal component analysis (PCA) illustrating the clustering of the five analyzed groups. **(C)** Heatmap visualizing the expression of 2,433 enriched genes in retinal microglia compared with retinal tissue (see **Figure 1B** top), grouped by genes that were also overexpressed in retinal microglia compared with all three subtypes of monocytes (n = 249, top of the heatmap), as well as those transcripts that were expressed at comparable levels in retinal microglia and at least one (n = 678, center of the heatmap) or all three subtypes of monocytes (n = 1,506, bottom of the heatmap). Groups and demographics are color-coded in the heatmap annotation at the top (see legend on the right). **(D)** Dotplots visualizing the top five Gene ontology (GO) biological processes (BP) and molecular functions (MF), which the enriched genes in retinal microglia (rMG-enriched from C) were involved in. The GeneRatio represents the proportion of associated genes to the total number of enriched genes. The adjusted p-value of each GO term is shown by color. **(E)** Network diagram visualizing the rMG-enriched genes associated with the most significantly enriched GO biological processes and molecular functions. The color represents mean expression in rMG of each gene. **(F)** Bar graphs illustrating the top five expressed genes in rMG associated to four of the most significantly enriched GO biological processes and molecular functions in rMG. The expression is also visualized for all three subtypes of monocytes as well as for retinal tissue. The lengths of the bars correspond to mean expression, while the black dots visualize expression in each sample.

To further define retinal microglia-enriched genes, the microglia gene signature (**Figure 1B**) was subsequently compared with the expression profiles of classical (CD45^+^CD11b^+^CX_3_CR1^+^CD14^++^CD16^-^), intermediate (CD45^+^CD11b^+^CX_3_CR1^+^CD14^++^CD16^+^), and non-classical monocytes (CD45^+^CD11b^+^CX_3_CR1^+^CD14^-^CD16^++^) (**Figure 2A**). Unsupervised cluster analysis using principal component analysis (PCA) revealed considerable differences between all four immune cell populations and an even more pronounced discrepancy between the immune cell populations and whole retinal tissue (**Figure 2B**). From a total of 2,433 transcripts in the microglia gene signature (**Figure 1B**), 2,184 genes were similarly expressed in comparison to monocytes (**Figure 2C**). However, 249 genes, such as *SPP1*, *C3*, and *C1QB* (Complement C1q B Chain), were identified to be enriched in comparison with all three monocyte subtypes (**Figure 2C** and **Supplementary Table 1**). In addition, published scRNA-Seq data of human retinal tissue (37) were reanalyzed to determine which of these 249 genes were specifically expressed in retinal microglia compared to 17 other retinal cell types. Only 76.7% (n = 191) of these 249 genes were detected by scRNA-Seq in any retinal cell type, whereas 82 genes were identified to be specifically expressed in retinal microglia compared to all other retinal cell types, among them *SPP1*, *C3*, *C1QB*, *TGFBR1*, and *TREM2* (**Supplementary Table 1**).

GO analysis demonstrated that the 249 retinal microglia-enriched transcripts that distinguish them from monocytes were most significantly enriched in biological processes such as chemotaxis (GO:0006935), defense response (GO:0031347), leukocyte activation (GO:0002694), secretion by cell (GO:1903530) and inflammatory response (GO:0050727) as well as molecular functions such as receptor ligand activity (GO:0048018), receptor regulator activity (GO:0030545), cytokine activity (GO:0005125), cytokine receptor binding (GO:0005126) and purinergic nucleotide receptor activity (GO:0045028) (**Figure 2D**). The contribution of each gene to these processes is visualized in the network diagram in **Figure 2E**, revealing that genes such as *IL1B* (Interleukin 1 Beta), *CCL2* (C-C Motif Chemokine Ligand 2), *CCL8* (C-C Motif Chemokine Ligand 8), *TREM2*, *CD84* (CD84 Molecule), *SPP1, C3* and *TGFBR1* act as retinal microglia-enriched key contributors to these processes. Interestingly, reanalysis of scRNA-Seq data (37) revealed that 13 of 19 expressed retinal microglia-enriched key genes were specific for retinal microglia, including all key factors mentioned above (**Supplementary Figure 3**). The expression of these and other top expressed microglia-enriched genes in comparison to all three subtypes of monocytes and whole retinal tissue are visualized in **Figure 2F**.

### Comparison of Human and Mouse Retinal Microglia Transcriptome

**Figure 3:**
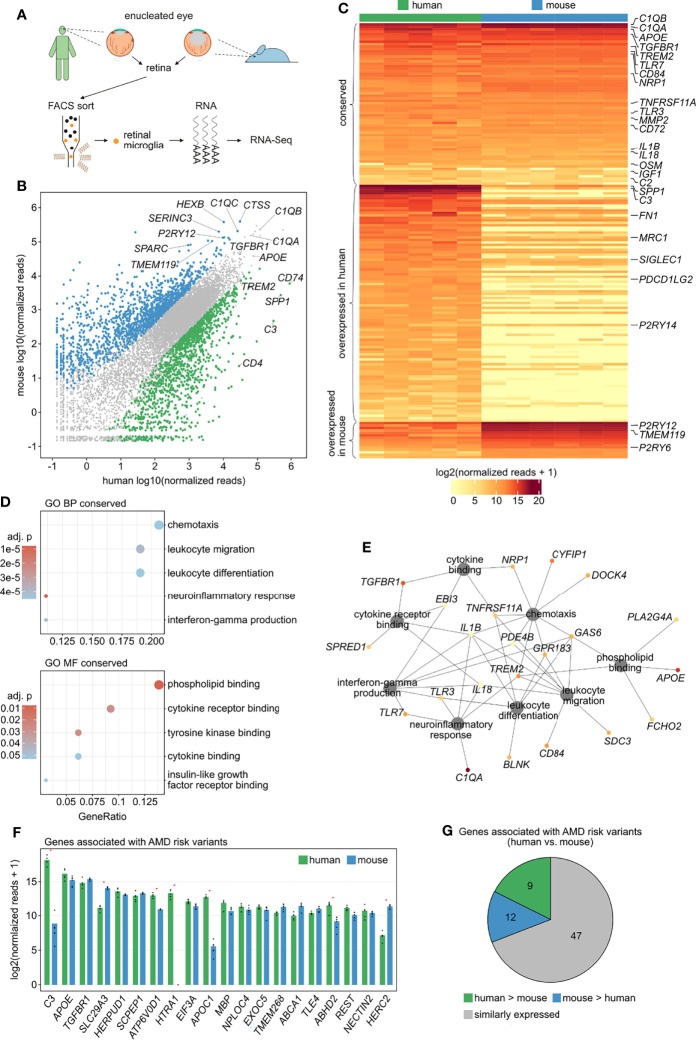
Comparison of human and murine retinal microglia transcriptional profiles. **(A)** Experimental setup: retinal microglia were FACS-isolated from five human enucleated eyes (CD45^+^CD11b^+^CX_3_CR1^+^MatMac^+^CCR2^-^) as well as from enucleated eyes from six *Cx3cr1* ^GFP/+^ mice (CD45^low^CD11b^+^CX_3_CR1^+^Ly6C^-^Ly6G^-^) followed by RNA sequencing. **(B)** Read plot visualizing expression levels of 11822 one-to-one orthologous genes in human and mouse retinal microglia highlighting differentially expressed genes in human (green) and mouse (blue) (log2FC > 2, adjusted p < 0.05). **(C)** Heatmap of the expression of retinal microglia-enriched genes identified in human (**Figure 2C**) compared with mice, with an one-to-one orthologous gene found for 178 of the 249 genes. Transcripts with comparable expression between human and mouse are provided as conserved factors at the top of the heatmap (n = 66). Overexpressed factors in human (n = 97) and mouse (n = 15) are visualized in the center and at the bottom of the heatmap, respectively (log2FC > 2, adjusted p < 0.05). **(D)** Dotplots visualizing the top five Gene ontology (GO) biological processes (BP) and molecular functions (MF), which the conserved genes in retinal microglia were involved in. The GeneRatio represents the proportion of associated genes to the total number of conserved genes. The adjusted p-value of each GO term is shown by color. **(E)** Network diagram visualizing the conserved genes associated with the most significantly enriched GO biological processes and molecular functions. The color represents mean expression of each gene in human and mouse (legend in C). **(F)** Bar graphs visualizing the expression levels of the 20 highest expressed genes associated with AMD risk variants in human and mouse retinal microglia. Red asterisks indicate differentially expressed genes (log2FC > 2, adjusted p < 0.05). **(G)** Pie chart illustrating relative expression of genes associated with AMD risk variants in human and mouse retinal microglia.

Studies in mice are considered a valuable tool for studying the biology of retinal microglia and for validation of translational approaches. However, to date, the extent to which mouse and human microglia differ, rendering mouse retinal microglia a valid model, is unknown. To fill this knowledge gap, we next compared the transcriptional profiles of human and mouse retinal microglia. To this end, the expression profiles of human retinal microglia described above were compared with those of murine retinal microglia FACS-isolated from six at least two years old *Cx3cr1* ^GFP/+^ mice (CD45^low^CD11b^+^CX_3_CR1^+^Ly6C^-^ Ly6G^-^) (**Figure 3A**). With respect to the 11,822 detected one-to-one orthologous genes, 75.8% (n = 8,965) were expressed at comparable levels (log2FC ≤ 2 and adjusted p ≥ 0.05) in both humans and mice (**Figure 3B**, grey), with genes such as *ACTB*, *PSAP*, *CSF1R* (Colony Stimulating Factor 1 Receptor), *C1QB* and *ITM2B* (Integral Membrane Protein 2B) representing the top five expressed conserved genes. However, several species-specific microglia genes were identified, with 1,405 transcripts (11.9%) being preferentially expressed in human retinal microglia (**Figure 3B**, green) and 1,452 genes (12.3%) being enriched in mice (**Figure 3B**, blue). Examples of genes predominantly expressed in human microglia were *CD74*, *SPP1* and *C3*. In contrast, genes preferably expressed in murine retinal microglia included *Ctss* (Cathepsin S), *Hexb* (Hexosaminidase Subunit Beta), *Serinc3* (Serine Incorporator 3) and *Sparc* (Secreted Protein Acidic And Cysteine Rich) (**Figure 3B**).

Expression of the microglia-enriched genes identified in humans (**Figure 2C**) was then compared with mice, which revealed 66 species-conserved retinal microglia-enriched transcripts, including *C1QA* (Complement C1q A Chain), *C2* (Complement C2), *APOE* (Apolipoprotein E), *TGFBR1*, *TREM2*, *NRP1* (Neuropilin 1) and *TNFRSF11A* (TNF Receptor Superfamily Member 11a), also known as RANK (**Figure 3C** and **Supplementary Table 2**). Nevertheless, there were several microglia-enriched factors which were predominantly expressed in human, among them *SPP1*, *C3* and *FN1* (Fibronectin 1), or murine retinal microglia, including *P2ry12*, *Tmem119* and *P2ry6* (Pyrimidinergic Receptor P2Y6) (**Figure 3C**). GO analysis revealed that the 66 conserved microglia transcripts were most significantly associated with biological processes such as chemotaxis (GO:0006935), leukocyte migration (GO:0050900), leukocyte differentiation (GO:0002521), neuroinflammatory response (GO:0150076) and interferon-gamma production (GO:0032609) as well as molecular functions including phospholipid binding (GO:0005543), cytokine receptor binding (GO:0005126), tyrosine kinase binding (GO:1990782), cytokine binding (GO:0019955) and insulin-like growth factor receptor binding (GO:0005159) (**Figure 3D**).

The connection of each gene to these processes is visualized in the network diagram in **Figure 3E**, providing the functionally most important conserved retinal microglia genes, including *IL1B*, *GAS6* (Growth Arrest Specific 6), *TREM2*, *TNFRSF11A*, *IL18* (Interleukin 18), *PDE4B* (Phosphodiesterase 4B), *GPR183* (G Protein-Coupled Receptor 183), *TLR3* (Toll Like Receptor 3), *TLR7* (Toll Like Receptor 7) and *TGFBR1*.

Comparing the expression of AMD-associated genes (9, 39) between human and mouse retinal microglia determined that the majority (69.1%) was expressed at comparable levels between both species, with, however 13.3% and 17.6% of genes being preferentially expressed in human or mouse, respectively (**Figure 3G**). Similarly, 65.0% of the 20 highest expressed AMD-related risk genes were comparably expressed between human and mice, among them *APOE*, *TGFBR1*, *HERPUD1* (Homocysteine Inducible ER Protein With Ubiquitin Like Domain 1), *SCPEP1* (Serine Carboxypeptidase 1) and *EIF3A* (Eukaryotic Translation Initiation Factor 3 Subunit A) (**Figure 3F**). However, transcripts such as *C3*, *ATP6V0D1* (ATPase H+ Transporting V0 Subunit D1), *HTRA1* (HtrA Serine Peptidase 1), *APOC1* (Apolipoprotein C1) and *ABHD2* (Abhydrolase Domain Containing 2, Acylglycerol Lipase) were significantly enriched in human microglia, whereas *Slc29a3* and *Herc2* (HECT and RLD domain containing E3 ubiquitin protein ligase 2) were enriched in mice (**Figure 3F**). DR-associated genes exhibited a similar distribution between human and mice retinal microglia. 65.0% of the 20 top expressed DR-related risk genes were comparably expressed, among them *PLXDC2*, *TCF4* (Transcription Factor 4), *SEC11C* (SEC11 Homolog C, Signal Peptidase Complex Subunit), *TBC1D5* (TBC1 Domain Family Member 5) and *USP7* (Ubiquitin Specific Peptidase 7). In contrast, transcripts such as *CPM* (Carboxypeptidase M), *MAN2A1* (Mannosidase Alpha Class 2A Member 1) and *UTRN* (Utrophin) were enriched in human and *Inpp4b* (Inositol Polyphosphate-4-Phosphatase Type II B) and *Slc16a7* (Solute Carrier Family 16 Member 7) in mice (**Supplementary Figure 4**).

## Discussion

As the tissue-resident macrophages of the CNS, microglia participate in critical processes like organ development and tissue homeostasis under healthy conditions, whereas microglial dysfunction is linked to the pathogenesis of various brain and retinal diseases, including Alzheimer’s disease, Parkinson’s disease and multiple sclerosis (1, 3–5, 9–11) as well as DR (12) and AMD (7, 8). While most of our knowledge about microglia is based on animal experiments, much less is known about human microglia, especially in the retina (10, 36). To advance our understanding of human retinal microglia, the present study applied fluorescence-activated cell sorting and RNA sequencing to characterize their transcriptional profile and to compare it with the one of whole retinal tissue, classical, intermediate and non-classical monocytes, as well as murine retinal microglia. The results provide novel in-depth insights into the molecular profile of human retinal microglia and advance our understanding of their role in human retinal homeostasis and diseases such as AMD or DR, offering foundations for potential immunomodulatory therapeutic approaches. Since most of our current knowledge on retinal microglia is based on animal models, this study additionally sheds light on the analogies between humans and mice, laying the foundation for future translational studies.

The generated transcriptional profiles revealed high quality and specificity parameters, as indicated by the absence of significant patient-, age- or sex-dependent variations, high cell- and tissue-specificity based on known cell-specific marker genes, and a low proportion of contaminating cells. Comparing the transcriptional profiles of retinal microglia with whole retinal tissue identified a retinal microglia gene signature, with genes such as *CD74*, *ACTB*, *SPP1, FTL* and *PSAP* being among the top expressed transcripts. Interestingly, a strong consistency compared to the top expressed genes in brain microglia was observed (9, 35), with genes such as *CD74*, *SPP1*, *ACTB*, *FTL* and *C3* yielding the highest concordance. However, there were also some genes whose expression differed in retinal and brain microglia, including *CD38* and *CD52*, highly expressed in retinal microglia, and *DOK6* and *DOCK3* enriched in brain microglia. Overexpression in retinal microglia suggests that *CD38* and *CD52*, known mediators of immunomodulation, neurodegeneration, and neuroinflammation in the brain (42, 43), may represent potential new therapeutic targets not only in the brain but also for degenerative and inflammatory diseases in the retina. *DOK6* and *DOCK3*, on the other hand, are both involved in neurite outgrowth (44, 45), indicating that microglia can modulate their expression profile adapted to the specific needs of their environment. Transcriptional profiling of retinal microglia in conjunction with parallel whole retina sequencing enabled the analysis of relative expression levels of disease-associated genes. Interestingly, several of the AMD- (e.g. *C3*, *APOE* and *TGFBR1*) and DR-associated risk genes (e.g. *PLXDC2* and *ARHGAP22*) were preferentially expressed in retinal microglia, which may indicate a role of retinal microglia in disease occurrence and progression.

Subsequently, the microglia gene signature was compared with classical, intermediate, and non-classical monocytes, revealing 249 retinal microglia-enriched genes. Interestingly, reanalysis of scRNA-Seq data of human retinal tissue (37) revealed that only 76.7% (n = 191) of these 249 genes were detected by scRNA-Seq in any retinal cell type, which might be explained by significantly higher sequencing depth as well as significantly higher numbers of detected genes in retinal microglia in the present study in comparison to the scRNA-Seq data. The 249 retinal microglia-enriched transcripts were found to be involved in cell-type related processes, such as chemotaxis, inflammatory response and cytokine activity, with a network analysis identifying genes such as *TREM2*, *P2RY12*, *IL1B*, *C3, TGFBR1* and *SPP1* being key factors in retinal microglia biology. Interestingly, reanalysis of scRNA-Seq data of human retinal tissue (37) confirmed that, among others, these genes were microglia-specific compared to 17 other retinal cell types. *TREM2* and *P2RY12* are well-established markers of both brain (9) and retinal microglia (36). Interleukin-1β and Interleukin-18, which also emerged among the retinal microglia-enriched factors, are two inflammatory cytokines released by activated inflammasomes in brain microglia (46) and were suggested to play a role in retinal neurodegenerative disease, such as AMD (47). SPP1, also known as Osteopontin, is an extracellular structural protein which is involved in the interaction between the innate and adaptive immune system (48) and plays a role in both inflammatory and degenerative processes in the central nervous system, as seen in multiple sclerosis and Alzheimer’s disease (49, 50). In the eye, *SPP1* expressed by retinal microglia was recently identified as a key mediator of retinal inflammation in the mouse model of choroidal neovascularization (CNV) and detected in human CNV (7). Furthermore, SPP1 expression was reported to be increased in monocyte-derived macrophages in AMD patients carrying the 10q26 risk haplotype and to be regulated by HTRA1 and CD47 signaling. In murine models of subretinal inflammation and AMD, SPP1 deletion or pharmacological inhibition reversed HTRA1-induced pathogenic persistence of mononuclear phagocytes in the subretinal space thus paving the way for SPP1 inhibitors for the treatment of AMD (50). In addition, several components of the complement system, including *C1q*, *C2*, and *C3*, were among the retinal microglia-enriched transcripts, indicating retinal microglia as mediators of complement activation. Since the complement system is known to be involved in the pathophysiology of AMD (39, 51, 52) and DR (53), these results provide further evidence for a possible pathophysiological involvement of retinal microglia.

Comparing the retinal microglia transcriptome between humans and mice resulted in a high proportion of conserved factors, which is in line with the recent identification of a cross-species conserved microglia core signature (54). However, about twelve percent of genes were overexpressed in humans or mice, respectively. Interestingly, these species-specific factors exhibited high consistency with those found in brain microglia (9). Similarly, analyzing the expression of AMD- and DR-associated risk genes in both species revealed that the majority of risk genes, were similarly expressed in both species, among them *APOE* and *TGFBR1*, which are established risk genes for AMD, as well as *PLXDC2* and *ARHGAP22* representing risk genes for DR (39). Consequently, murine studies regarding the role of *APOE* and *TGFBR1*, as well as *PLXDC2* and *ARHGAP22* in microglia in AMD and DR seem to represent a suitable model for the human situation. However, the results also revealed that some AMD- and DR-risk genes such as *HTRA1* and *CPM* are rarely expressed in murine retinal microglia. Therefore, investigating these retinal microglia factors in the context of AMD and DR in the mouse model is presumably less useful. Taken together, these findings improve our understanding of the similarities and differences of retinal microglia between humans and mice and will facilitate the transferability of the findings from the murine to the human situation.

We acknowledge that this study is limited by the use of melanoma eyes, which does not fully exclude the possibility of retinal microglia being activated or affected by the tumor environment. However, comparing the transcriptional profiles of vitreous macrophages (hyalocytes), which were also isolated from these melanoma eyes but not analyzed in this study, with vitreous macrophages isolated during vitrectomy for macular pucker or macular hole (19), revealed a high degree of similarity (data not shown), which argues against a significant melanoma-related affection in our study. However, general and melanoma-independent activation of vitreous macrophages and retinal microglia cannot completely be excluded. In addition, caution should be exercised in our methodological approach of comparing isolated microglia with gene expression in the whole retina. Most of the genes mentioned are also expressed in other cells of the retina, such as photoreceptors, or glia cells, where they are present at high levels and thus could overshadow gene expression in other cells. However, the high agreement of our data with data from scRNA-Seq studies (37, 38) justifies our approach, which should be validated in the future by further scRNA-Seq analyses to compare expression in different cell types. Finally, for the comparison between human and murine microglia, mice aged at least 2 years were investigated resembling the average age of the patients that were included in this study. Since the age has a considerable influence on the expression profile of retinal microglia (18), discrepancies must be taken into account when investigating younger mice. A strength of this study is the use of fresh tissue, which was processed instantly after enucleation, allowing to preserve the RNA of sorted cells within four hours after surgery, which is significantly faster compared to other studies using post-mortem tissue with a time from death to preservation of 8 up to 17 h (36, 38). This advantage is of particular relevance considering that retinal tissue can provide stable RNA only if processed within five hours (55).

In summary, the present study applied fluorescence-activated cell sorting and RNA sequencing to characterize the human retinal microglia transcriptome and to compare it with that of classical, intermediate and non-classical monocytes, as well as murine retinal microglia. These data provide detailed insights into the molecular profile of human retinal microglia and indicate a high similarity to brain microglia. Also, we show that several risk genes for AMD or DR, which may contribute to disease occurrence and progression, are expressed in retinal microglia. Finally, we found a high degree of similarly expressed genes between humans and mice supporting the suitability of murine studies to understand human retinal microglia functions.

## Data Availability Statement

The original contributions presented in the study are publicly available. This data can be found here: https://www.ncbi.nlm.nih.gov/geo/query/acc.cgi?acc=GSE193161.

## Ethics Statement

The studies involving human participants were reviewed and approved by ethics committee (University Freiburg, Germany, approval number 20-1165). The patients/participants provided their written informed consent to participate in this study. The animal study was reviewed and approved by animal care and use committee, University Freiburg, Germany.

## Author Contributions

JW: designing research studies, analyzing RNA sequencing data, designing the figures, writing the original draft of the manuscript. SB: designing research studies, conducting experiments, review and editing the manuscript. D-DR: review and editing the manuscript. HA: supervising experiments, review and editing the manuscript. GS: supervising experiments, review and editing the manuscript. PW: review and editing the manuscript. AS: designing research studies, conducting experiments, review and editing the manuscript. CL: designing research studies, supervising experiments, review and editing the manuscript. All authors contributed to the article and approved the submitted version.

## Conflict of Interest

The authors declare that the research was conducted in the absence of any commercial or financial relationships that could be construed as a potential conflict of interest.

## Publisher’s Note

All claims expressed in this article are solely those of the authors and do not necessarily represent those of their affiliated organizations, or those of the publisher, the editors and the reviewers. Any product that may be evaluated in this article, or claim that may be made by its manufacturer, is not guaranteed or endorsed by the publisher.
