# Supplementary material for: In-Depth Molecular Profiling Specifies Human Retinal Microglia Identity
Source: Front Immunol. 2022 Mar 18;13:863158. doi: 10.3389/fimmu.2022.863158 (PMC8971200; doi:10.3389/fimmu.2022.863158)
Supplement: Supplementary file 1 [file DataSheet_1.pdf]

Supplement

In-depth molecular profiling specifies human retinal microglia identity

Julian Wolf[1], Stefaniya Boneva[1], Dennis-Dominik Rosmus[2], Hansjürgen Agostini[1], Günther Schlunck[1], Peter Wieghofer[2,3], Anja Schlecht[1,4*] and Clemens Lange[1,5*]

[1] Eye Center, Medical Center, Faculty of Medicine, University of Freiburg, Germany

[2] Institute of Anatomy, Leipzig University, Leipzig, Germany

[3] Cellular Neuroanatomy, Institute of Theoretical Medicine, Medical Faculty, University of Augsburg, Augsburg, Germany

[4] Institute of Anatomy, Wuerzburg University, Wuerzburg, Germany

[5] Ophtha-Lab, Department of Ophthalmology at St. Franziskus Hospital, Muenster, Germany

[*] contributed equally as senior authors

Corresponding author: Clemens Lange MD PhD, Ophtha-Lab, Department of Ophthalmology at St. Franziskus Hospital, Hohenzollernring 74, 48145 Muenster, Germany, tel. +49 251 9352727, fax +49 251 9351111, e-mail: clemens.lange@augen-franziskus.de

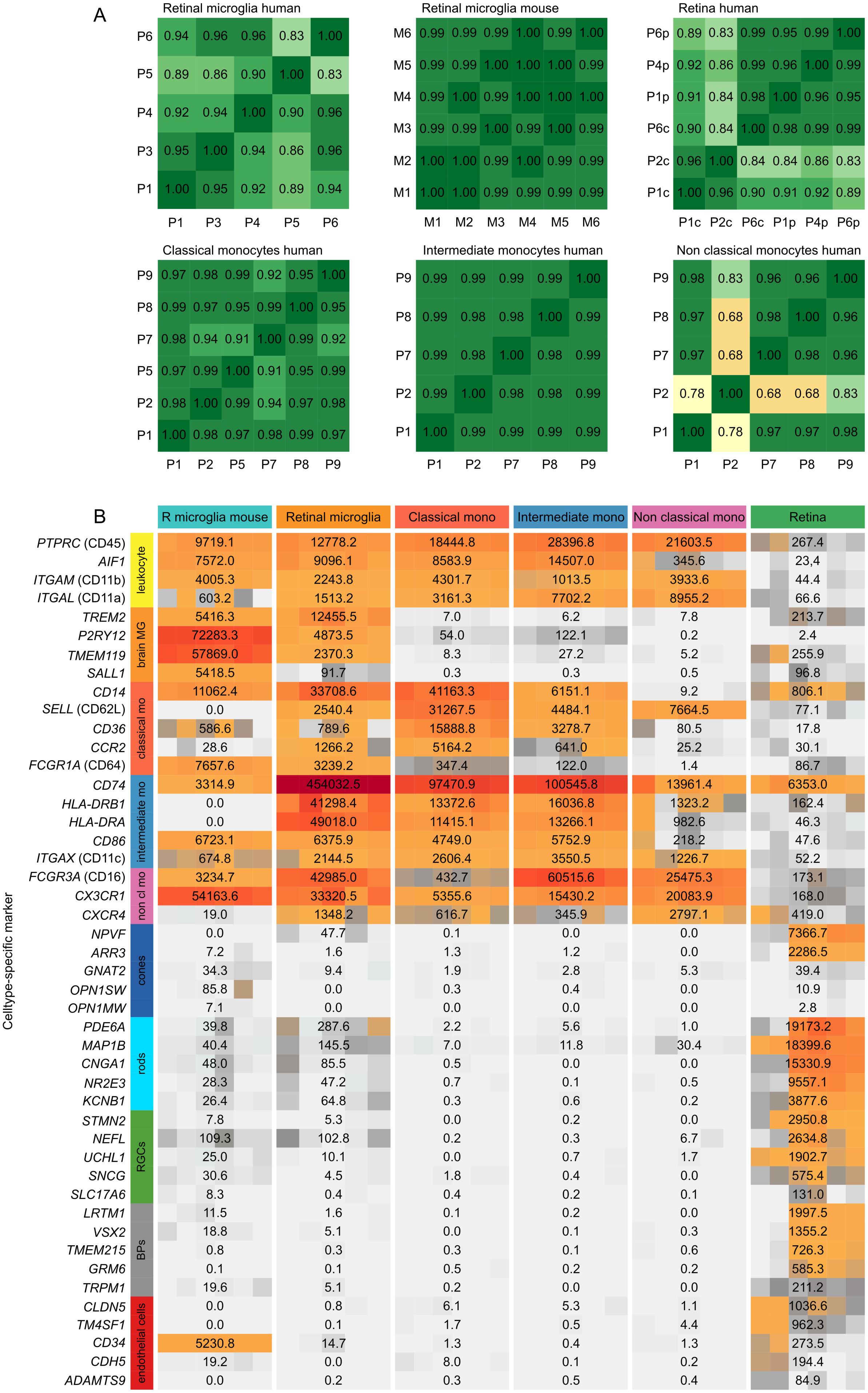

Supplementary figure 1: Quality and purity check. (A): Correlation plots visualizing Pearson correlation coefficients between any two patients (P) or mice (M) for all cell populations or tissue types (c = centre, p = periphery). (B): Expression of known celltype-specific marker genes in all 5 immune cell populations as well as in retinal tissue. Each column represents one group and each row one marker gene. Groups of marker genes for each celltype are visualized in the row annotation on the left. Numbers correspond to mean of normalized reads per group. Abbreviations: mo(no): monocyte, non cl: non classical, MG: microglia, R: retinal, RGC: retinal ganglion cell, BP: bipolar cell.

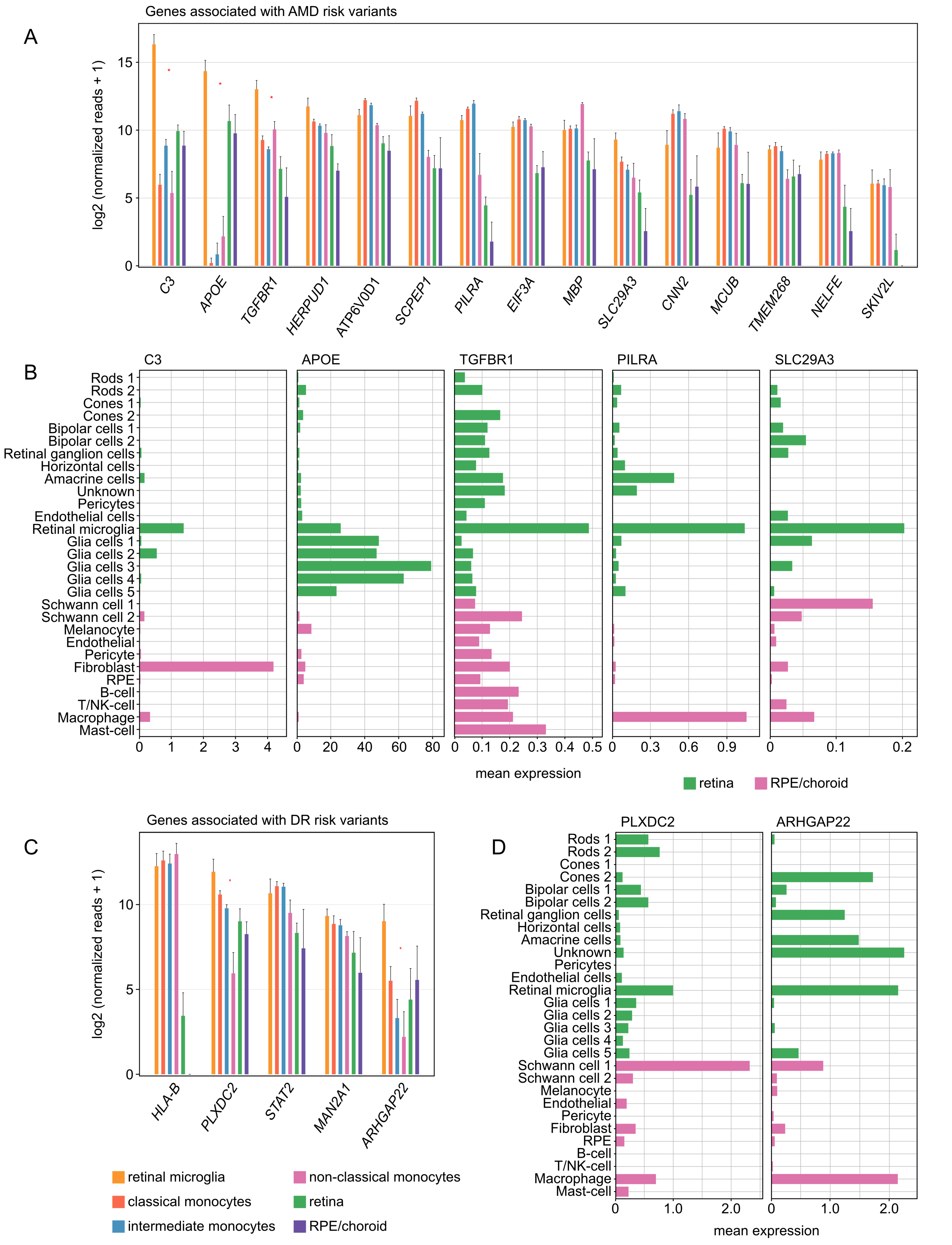

Supplementary figure 2: Bar graphs visualizing the expression levels of the 15 AMD (A) and 5 DR (C) risk genes identified to be overexpressed in retinal microglia when compared to retinal and RPE/choroid tissue (see Figure 1 F-G) in comparison to three subtypes of monocytes. Red asterisks indicate genes which were not only overexpressed in retinal microglia versus retinal and RPE/choroid tissue, but also when compared to all three subtypes of monocytes. (B+D) Expression of AMD (B) or DR (D) associated risk genes mainly expressed in retinal microglia compared to 28 other retinal and RPE/choroid cell types, as determined by reanalysis of published scRNA-Seq data of human retinal and RPE/choroid tissue.

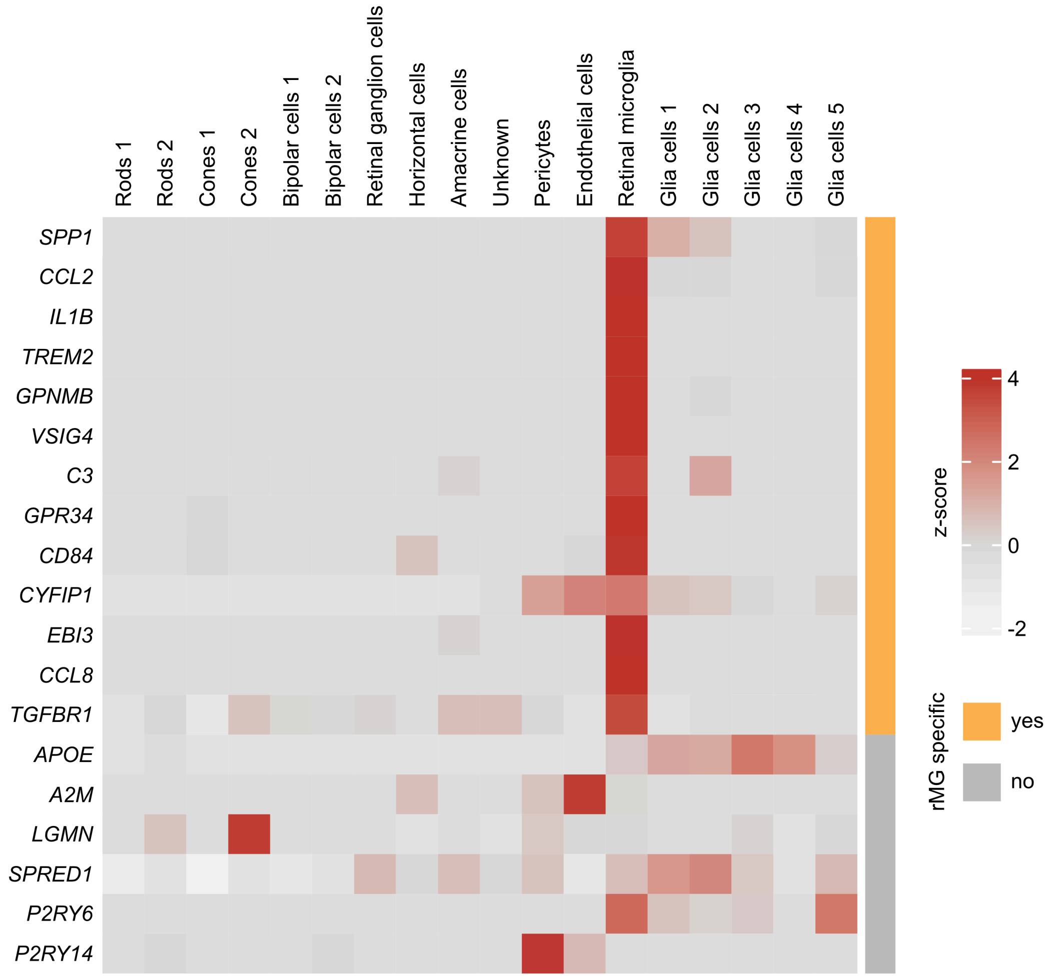

Supplementary figure 3: Heatmap visualizing expression of the 20 retinal microglia enriched genes identified in the network diagram in Figure 2E in published single cell RNA sequencing (scRNA-Seq) data of the human retina. 19 of these genes were detected by scRNA-Seq and 13 were specific for retinal microglia (yellow, see legend). The z-score represents a gene's expression in relation to its mean expression by standard deviation units.

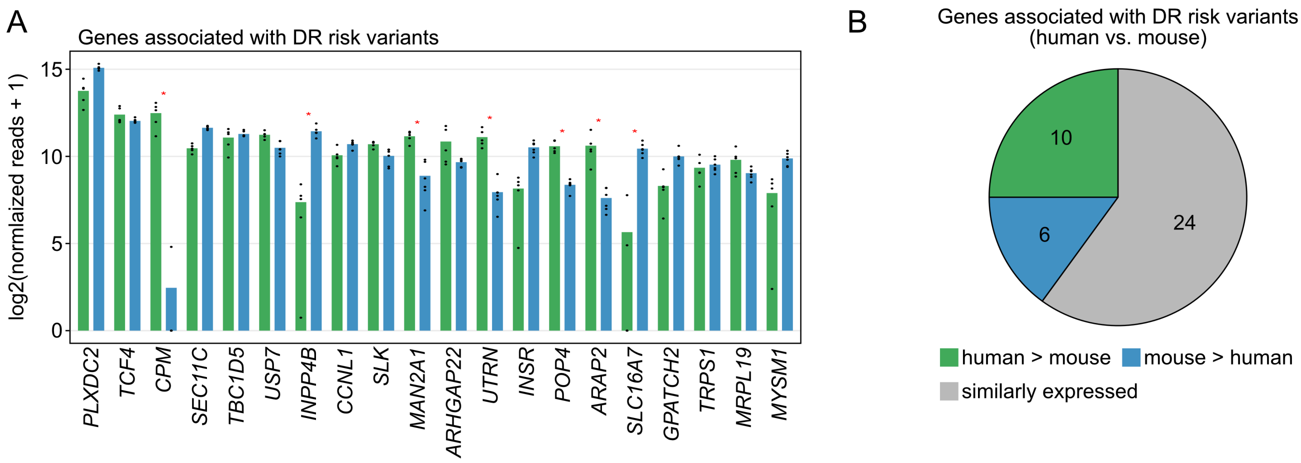

Supplementary figure 4: (A) Bar graphs visualizing the expression levels of the 20 highest expressed genes associated with DRP-risk variants in human and mouse retinal microglia. Red asterisks indicate differentially expressed genes (log2FC > 2, adjusted p < 0.05). (B) Pie chart illustrating relative expression of genes associated with DRP-risk variants in human and mouse retinal microglia.
